# Laboratory Exercise to Measure Plasmid Copy Number by qPCR

**DOI:** 10.1128/jmbe.00125-21

**Published:** 2021-07-30

**Authors:** Benjamin David, Jinbei Li, Faisal Masood, Caroline Blassick, Paul Jensen, Karin Jensen

**Affiliations:** a Department of Bioengineering, University of Illinois Urbana-Champaign, Urbana, Illinois, USA; b Carl R. Woese Institute for Genomic Biology, University of Illinois Urbana-Champaign, Urbana, Illinois, USA; c College of Medicine, University of Illinois at Chicago, Chicago, Illinois, USA; d Department of Biomedical Engineering, Boston University, Boston, Massachusetts, USA

**Keywords:** qPCR, laboratory instruction

## Abstract

Quantitative PCR (qPCR) has numerous applications in biology. In an educational setting, qPCR provides students an opportunity to better understand the PCR mechanism by providing both quantitative information about the reactions and also data to troubleshoot PCRs (e.g., melt curves). Here, we present a relatively short (2-h) laboratory activity to demonstrate qPCR to quantify plasmid copy number (CN) by measuring the cycle threshold (*C_T_*) values for a genomic gene and a plasmid gene using transformed cells as a template. The activity can be combined with additional laboratory exercises, including bacterial transformation, to create the template to be used in the qPCRs. This lab activity is ideal for undergraduate laboratory courses that include recombinant DNA technology.

(This work was presented at the 2020 Biomedical Engineering Society annual meeting).

## INTRODUCTION

PCR is one of the most foundational tools in molecular bioengineering and has become indispensable in many disease diagnostic methods, such as COVID-19 testing (1). Quantitative PCR (qPCR) adds real-time monitoring of the PCR amplification process to give information about the template abundance. Template abundance can be used to determine the expression level of different genes, the severity of an infection, or the copy number (CN) of a plasmid. In educational laboratory settings, the study of qPCR can enable students to closely examine and better understand the PCR mechanism from a quantitative perspective, improving on activities geared only toward product detection by traditional PCR (2, 3). While there are other reported lab activities that involve qPCR, they are either limited to using qPCR for detection, which does not necessitate quantitative analysis (4, 5), or require RNA extraction and quantification which may be challenging and irrelevant for a second-year undergraduate lab course (6–11). Here, we describe a 2-h lab exercise using qPCR to quantify CN of the pGLO plasmid in Escherichia coli strain DH5α. Cycle thresholds (*C_T_*) are measured for both a chromosomal and plasmid gene, which are then used to calculate the plasmid CN. This exercise addresses some limitations to using qPCR in lab courses by reducing the time, complexity, and cost necessary to complete the activity.

## PROCEDURE

### Equipment

A real-time PCR machine is required for this exercise. Two pairs of primers are used, one pair for the pGLO plasmid gene *bla*, the other one for an E. coli DH5α genomic gene *alaA* (Table 1). The genomic gene *alaA* was selected due to its location opposite the origin of replication (*ori*) on the bacterial chromosome, which minimizes the risk of overestimating genomic DNA abundance in cells undergoing multiple simultaneous rounds of chromosome replication. Luna Universal qPCR Master Mix from New England BioLabs is used for the reactions. A complete list of materials is included in Appendix 1.

**TABLE 1:** qPCR primers

Gene	Primer	Sequence	Product length	Predicted melt temp
*alaA*	F1	ACACGCCAAAGGCTACATCG	139	77.4°C
	R1	ACGACCGCCAGGGGTAATAA		
*bla*	F1	ATTATCCCGTGTTGACGCCG	198	76.8°C
	R1	TTCGGTCCTCCGATCGTTGT		

### Instructor preparation

The template used for this experiment is E. coli DH5α cells transformed with pGLO plasmid (Bio-Rad). Transformed cells can be prepared by the instructor or can be generated by students in a laboratory session preceding the session described here. Instructors also prepare primer stocks and aliquots of qPCR Master Mix ahead of the laboratory session. Complete instructions for instructors are provided in Appendix 1 in the supplemental material.

### Laboratory session

**FIG 1:**
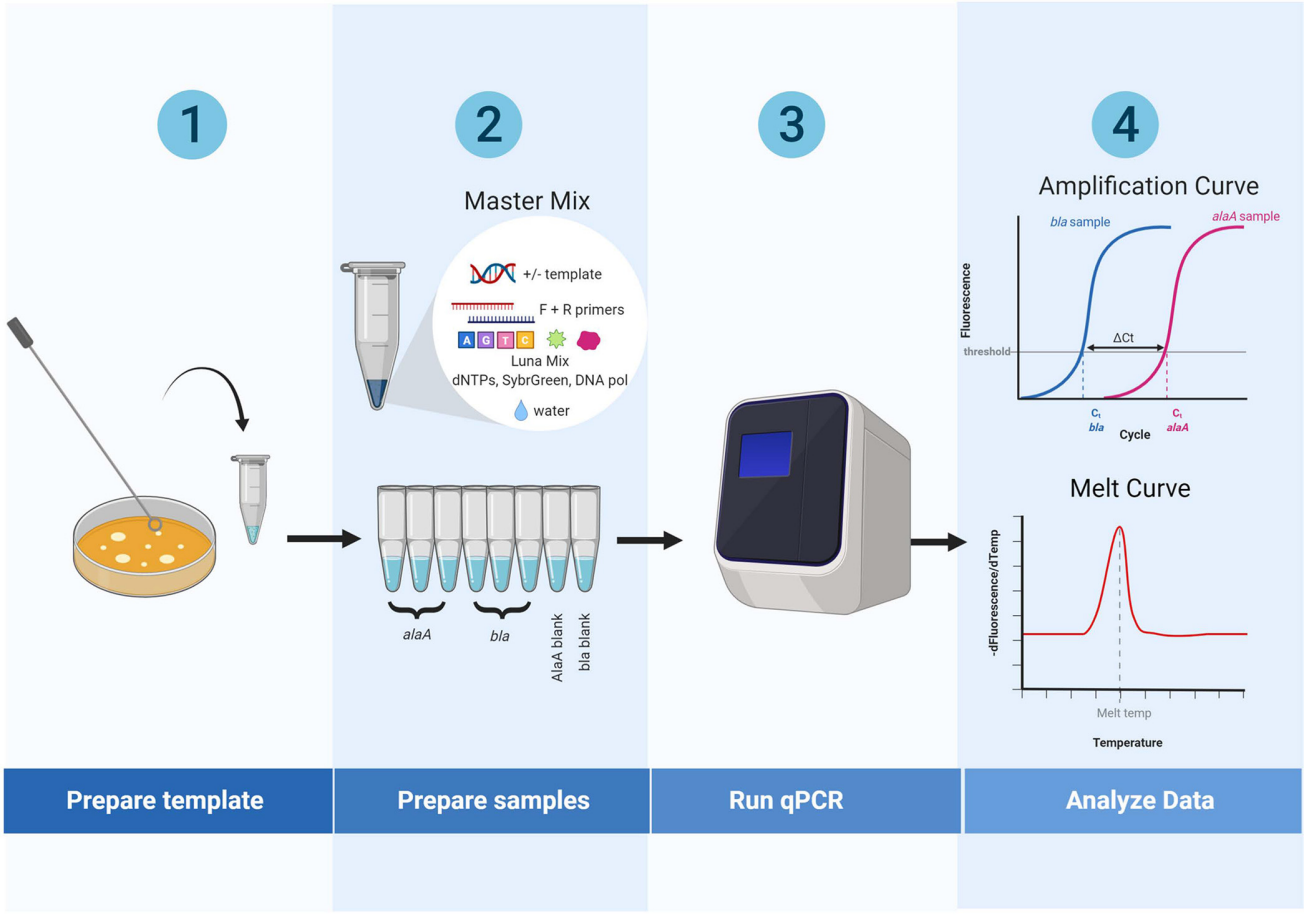
Laboratory exercise overview.

**FIG 2:**
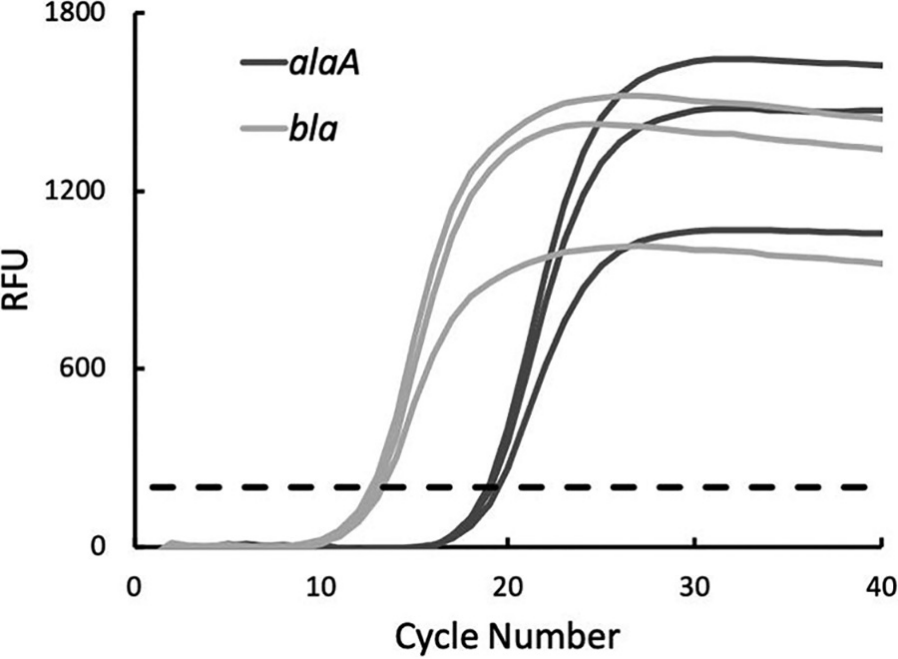
Amplification curves.

**FIG 3:**
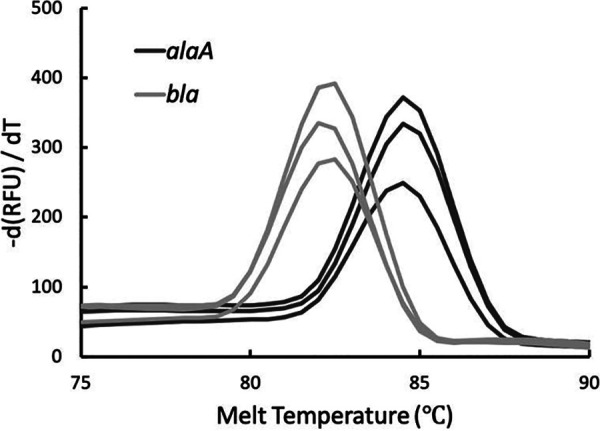
Melt curves.

**FIG 4:**
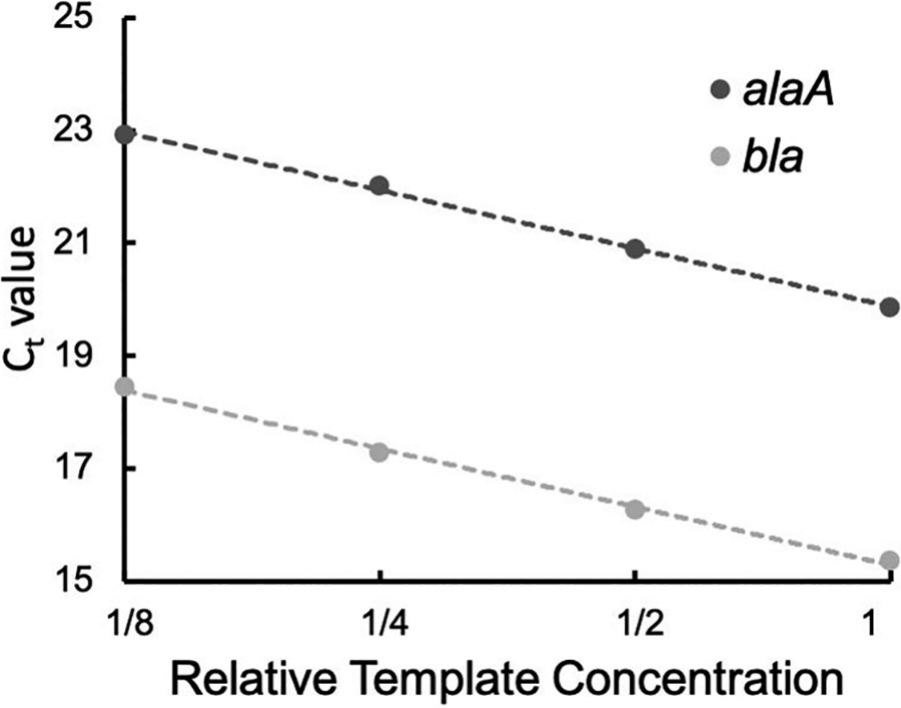
qPCR primer efficiency.

The protocol was optimized for colony PCR, eliminating the need for DNA extraction steps (Fig. 1). One colony of E. coli DH5α cells transformed with the pGLO plasmid is picked and suspended in 25 μl of water as the template. The following reactions are set up in triplicate for the genomic gene and plasmid gene separately: water, forward and reverse primers, qPCR Luna mix, and template. Two nontemplated reactions are used to validate the specificity of each primer set. The SYBR/FAM channel is chosen for detection. The experiment generates *C_T_* values for each reaction. Average *C_T_* values are used to calculate the plasmid CN by students: CN = 2^-(*C_T_*_plasmid – *C_T_*_genomic) (12). The amplification process can also be visualized through the amplification curves (Fig. 2). Melt curve analysis shows a single peak for the amplicon from every sample, indicating high primer specificity (Fig. 3). An amplification efficiency of 96% was determined for both *bla* and *alaA*, indicating a lack of bias in the CN quantification model based on starting template abundance (12) (Fig. 4). Taken together, these results validate the use of the qPCR primers designed for this activity. Through the exercise, students will be given an opportunity to study the qPCR process closely and understand its quantitative aspects, offering a broader perspective than performing traditional PCR.

### Safety issues

This laboratory exercise should be performed at biosafety level 1 (BSL1). Instructors should refer to the ASM BSL1 guidelines for laboratory space requirements, personal protection requirements, and standard laboratory practices. Caution should be used when handling ampicillin, as it can cause allergic reactions or irritation to the eyes, respiratory system, and skin. Manufacturer safety guidelines for individual reagents should be followed and safety datasheets for all reagents should be provided to students.

## RESULTS

The laboratory exercise described has been implemented for seven semesters, with approximately 300 students completing the activity. Students completing the laboratory exercise receive prior training in micropipetting and lectures on PCR and qPCR. Instructors observe that, on average, most students successfully generate *C_T_* values and quantify the pGLO CN. We found that measuring volumes less than 5 μl was difficult for some students, which can lead to variable amplification across replicates. This difficulty could be mitigated by preparing more dilute primer stocks and using larger volumes. Following data collection, students meet with the instructor to review data, including amplification and melt curves. Students are instructed on data processing to obtain the pGLO CN. Student assessments include a written laboratory report where students validate features and CN of the pGLO plasmid (13).

## DISCUSSION

Here, we describe a 2-h qPCR laboratory exercise to quantify the CN of a plasmid in E. coli. This activity does not require DNA or RNA extraction kits, reducing instructional costs; however, a limitation is the requirement of access to a real-time PCR machine. The activity is highly modular and could be combined with an additional lab activity where students first transform cells with the pGLO plasmid. Future work to improve this laboratory exercise should include assessments of student self-efficacy and understanding of qPCR concepts. This exercise is suited for teaching undergraduate lab topics, including PCR, qPCR, bacterial transformation, and biological measurements. Individual project extensions could include testing other plasmids or studying conditions that could affect plasmid CN. Further, the activity could be expanded to include topics of plasmid engineering, where modifications to plasmids (e.g., modifications of the origin of replication, protein expression load, or plasmid size) are evaluated with respect to how they impact the plasmid CN. The measurement of plasmid CN can also be coupled to measurements of fluorescent protein expression levels to showcase the effects of CN.
