# Supplementary material for: Laboratory Exercise to Measure Plasmid Copy Number by qPCR
Source: J Microbiol Biol Educ. 2021 Jul 30;22(2):e00125-21. doi: 10.1128/jmbe.00125-21 (PMC8442022; doi:10.1128/jmbe.00125-21)
Supplement: SUPPLEMENTAL FILE 1 — Download JMBE00125-21_Supp_1_seq2.docx, DOCX file, 0.02 MB [file jmbe00125-21_supp_1_seq2.docx]

**Appendix 1: Instructor preparation protocol**

**Equipment needed:**

qPCR machine

Bacteria incubator

Micropipettes

Water bath or heat block

**Materials needed:**

| 8-strip PCR Tubes | Biorad TLS0801 |
| --- | --- |
| Optical PCR Tube Lids | Biorad TCS0803 |
| 2x qPCR LunaMix | NEB #M3003S |
| Molecular Biology Grade Water | N/A |
| AlaA_F1 Primer (10 µM) | IDT |
| AlaA_R1 Primer (10 µM) | IDT |
| Bla_F1 Primer (10 µM) | IDT |
| Bla_R1 Primer (10 µM) | IDT |
| pGLO plasmid | Biorad 1660405EDU |
| DH5α competent cells* | NEB C29871 |
| LB agar plates with ampicillin* | N/A |

*Alternatively, the pGLO bacterial transformation kit sold by Biorad (product #1660003EDU can be used)

**Primers**

| **Gene** | **Primer** | **Sequence** | **Product length** | **Predicted Melt Temperature** |
| --- | --- | --- | --- | --- |
| *alaA* | F1 | ACACGCCAAAGGCTACATCG | 139 | 77.4°C |
|  | R1 | ACGACCGCCAGGGGTAATAA |  |  |
| *bla* | F1 | ATTATCCCGTGTTGACGCCG | 198 | 76.8°C |
|  | R1 | TTCGGTCCTCCGATCGTTGT |  |  |

**Instructor preparation:**

Aliquot 100 μL 2x qPCR Luna MasterMix (NEB #M3003S) per group.

Aliquot 200 μL Molecular biology grade (MBG) water per group.

Aliquot 5 μL of each 10 µM primer (AlaA_F1, AlaA_R1, Bla_F1, and Bla_R1) per group. Note that primers in the original tubes from IDT are 100 µM. You will have to create a 1:10 dilution in another tube before aliquoting. Dilute in MBG water. All stocks should avoid repeated freeze/thaws, including the master stock in the IDT tubes. These should be aliquoted for future use.

Transform *E. coli* with pGLO plasmid (note that this can be done by students in a prior lab session) and grow on LB plates with ampicillin. Directions are provided in the pGLO bacterial transformation kit (Biorad).

**Each lab group will need**:

*E. coli* transformed with pGLO plasmid

Strip of 8 PCR tubes (Biorad TLS0801)

Strip of **optical** PCR tube lids (ordinary lids will not allow proper measurement) (Biorad TCS0803)

100 μL 2x qPCR Luna MasterMix (NEB #M3003S)

200 μL Molecular biology grade water

5 μL alaA_F1 (10 µM)

5 μL alaA_R1 (10 µM)

5 μL bla_F1 (10 µM)

5 μL bla_R1 (10 µM)

**Appendix 2: Student protocol**

**Learning Objectives:** To learn how to experimentally determine the copy number of a plasmid using qPCR.

**Overview:** qPCR will be performed on bacteria colonies to assess the number of pGLO plasmids in each cell (copy number).

**Purpose:** The acquisition of cycle threshold (C_t_) data for a genomic gene (*alaA*) and a pGLO gene (*bla*) will allow the determination of the average copy number (CN) of pGLO plasmids in the cell. The plasmid extraction from the overnight culture will generate a plasmid stock of pGLO plasmid that could be used for future experiments.

**Background:** qPCR, or quantitative polymerase chain reaction, is a technique which can be used to quantify the amount of a target DNA molecule in a sample. It is also referred to as “real-time PCR,” because the amount of DNA in the reaction is continuously measured as the reaction proceeds. This measurement is done through the use of fluorescent dyes which intercalate (insert themselves between the strands of) all double-stranded DNA product. The amount of fluorescence read by the qPCR machine corresponds to the amount of DNA product formed. By comparing this value to that of a reference gene, we can determine the relative amount of the target DNA molecule in the initial sample. qPCR requires the careful design of primers, which are short (~20 nucleotide), single-stranded pieces of DNA that match the sequence of the DNA to be amplified. Primers serve as the starting point for the amplification of the target DNA sequence. In order to copy both sides of the DNA sequence, two primers are needed: one “forward” primer to copy the sequence from the beginning, and one “reverse” primer to copy the complementary sequence from the other side. Primers are designed such that they will resist annealing to each other, which forms double-stranded “primer dimers” that can throw off a qPCR measurement. In this lab we will be transforming a DNA plasmid and determining its copy number. After transformation, we will examine transformed and control cells to qualitatively determine the function of the plasmid. We will then quantitatively determine the copy number of the plasmid by performing qPCR on a gene from the plasmid in comparison to a reference gene on the *E. coli* chromosome.

**Safety Considerations:** *E. coli* strain DH5α is a nonpathogenic strain of *E. coli*. It has been genetically modified to only grow on enriched medium. Standard lab practices must be followed including proper surface decontamination, hand washing, PPE (long pants, closed toed shoes, lab coat, gloves), and waste disposal. These procedures will be reviewed at the start of the lab. Please ask your instructor/TA if you have any questions or concerns. Ampicillin can cause allergic reactions or irritation to the eyes, respiratory system, and skin. If ampicillin contacts your eyes, use the eye wash station to rinse your eyes with water for a minimum of five minutes and report the incident to your instructor/TA for subsequent reporting.

**Materials Needed:**

- Strip of 8 PCR tubes
- Strip of 8 optical PCR tube lids
- 100 μL 2x qPCR Luna Mix
- 100 μL Molecular biology grade water
- 5 μL alaA_F1 primer
- 5 μL alaA_R1 primer
- 5 μL bla_F1 primer
- 5 μL bla_R1 primer

**Protocol:**

1. Pick a single *E. coli* colony from the provided plate and suspend it in 5 μL of molecular biology grade water. This is your “template” DNA.
2. Dilute the template DNA 1:5 using more molecular biology grade water. Mix thoroughly.
   1. Diluting the template DNA prevents the reaction from amplifying too quickly.
3. Using filtered pipette tips, add the following components in the order provided. Set up this reaction in triplicate in the first 3 tubes of your 8-strip. Calculate a "master mix" to set up these reactions (i.e. mix the reagents together in a larger "batch" before dividing the mixture into 3 separate tubes). This step should be done before class and entered in your laboratory notebook. Each single reaction should contain:

7 μL molecular biology grade water

0.5 μL alaA_F1

0.5 μL alaA_R1

10 μL qPCR Luna mix (contains DNA polymerase, SYBR Green dye, and dNTPs)

2 μL template DNA

1. Repeat step 3 using the other two primers (bla_F1 and bla_R1) in tubes 4-6 of your 8-strip.
2. In tube 7 of your 8-strip, add the following components in the order provided. This serves as a negative control or "blank" for the *alaA* amplification.

9 μL molecular biology grade water

0.5 μL alaA_F1

0.5 μL alaA_R1

10 μL qPCR Luna mix

1. Repeat step 5 using the other two primers (bla_F1 and bla_R1) in the last tube of your 8-strip.
2. Place your 8-strip in the qPCR machine. Be sure to note the placement and orientation of your samples so you can identify your data later.

**Data Analysis:**

1. Review the melt curves for each sample. It is expected that the melt curves for *alaA* and *bla* samples show a single peak at 77.4 °C and 76.8 °C, the melting temperature of the expected amplicons, respectively. The blank samples, having no template added, are not expected to amplify and thus a “not detected” result is expected. Additional peaks or peaks at different temperatures than those expected for the amplicons may indicate contamination or primer dimers. Unexpected melt curves could be troubleshooted by running the sample on a gel or sequencing.
2. For each set of replicates (*alaA* and *bla*), calculate the average C_t_ value as determined from the amplification curves. It is expected that replicates for each amplicon are comparable (within 1-2 cycle thresholds). Large deviations of individual samples may indicate a problem with the experiment.
3. Using the average C_t_ values for *alaA* and *bla*, calculate the CN of pGLO plasmid using the formula: CN = 2^-(C_t__plasmid – C_t__genomic) where C_t__plasmid is the average value of the *bla* replicate C_t_ values and C_t__genomic is the average value of the *alaA* replicate C_t_ values.
